# The Cytoskeleton Effectors Rho-Kinase (ROCK) and Mammalian Diaphanous-Related (mDia) Formin Have Dynamic Roles in Tumor Microtube Formation in Invasive Glioblastoma Cells

**DOI:** 10.3390/cells11091559

**Published:** 2022-05-05

**Authors:** Kathryn N. Becker, Krista M. Pettee, Amanda Sugrue, Kevin A. Reinard, Jason L. Schroeder, Kathryn M. Eisenmann

**Affiliations:** 1 Department of Cell and Cancer Biology, University of Toledo Health Science Campus, Toledo, OH 43614, USA; kathryn.becker@rockets.utoledo.edu (K.N.B.); krista.pettee@utoledo.edu (K.M.P.); 2 Department of Chemistry and Biochemistry, University of Heidelberg, Tiffin, OH 44883, USA; sugrue@marshall.edu; 3 Division of Neurosurgery, ProMedica Toledo Hospital, Toledo, OH 43606, USA; kevin.reinardmd@promedica.org (K.A.R.); jason.schroeder5@utoledo.edu (J.L.S.); 4 Department of Surgery, University of Toledo Health Science Campus, Toledo, OH 43614, USA

**Keywords:** glioblastoma, invasion, tumor microtube, actin, mDia formin, Rho-kinase, cytoskeleton

## Abstract

Glioblastoma (GBM) is a progressive and lethal brain cancer. Malignant control of actin and microtubule cytoskeletal mechanics facilitates two major GBM therapeutic resistance strategies—diffuse invasion and tumor microtube network formation. Actin and microtubule reorganization is controlled by Rho-GTPases, which exert their effects through downstream effector protein activation, including Rho-associated kinases (ROCK) 1 and 2 and mammalian diaphanous-related (mDia) formins (mDia1, 2, and 3). Precise spatial and temporal balancing of the activity between these effectors dictates cell shape, adhesion turnover, and motility. Using small molecules targeting mDia, we demonstrated that global agonism (IMM02) was superior to antagonism (SMIFH2) as anti-invasion strategies in GBM spheroids. Here, we use *IDH*-wild-type GBM patient-derived cell models and a novel semi-adherent in vitro system to investigate the relationship between ROCK and mDia in invasion and tumor microtube networks. IMM02-mediated mDia agonism disrupts invasion in GBM patient-derived spheroid models, in part by inducing mDia expression loss and tumor microtube network collapse. Pharmacological disruption of ROCK prevented invasive cell-body movement away from GBM spheres, yet induced ultralong, phenotypically abnormal tumor microtube formation. Simultaneously targeting mDia and ROCK did not enhance the anti-invasive/-tumor microtube effects of IMM02. Our data reveal that targeting mDia is a viable GBM anti-invasion/-tumor microtube networking strategy, while ROCK inhibition is contraindicated.

## 1. Introduction

Glioblastoma (GBM) is a rapidly progressive and universally lethal brain cancer that represents nearly half of all primary malignant central nervous system (CNS) tumors [1]. The poor outcomes that characterize GBM are rooted in multiple interconnected mechanisms of therapeutic resistance that make this disease particularly difficult to effectively treat [2,3,4,5,6,7,8,9,10]. This multifactorial foundation of GBM therapy resistance suggests that a clinically meaningful improvement in GBM outcomes will require a therapeutic strategy that addresses each of these mechanisms. Consequently, therapeutic strategies that can singly address multiple molecular programs underlying GBM therapy resistance represent particularly high-value therapeutic targets.

Malignant control of cytoskeletal mechanics facilitates at least two major strategies of GBM therapeutic resistance—diffuse CNS invasion [11] and tumor microtube network formation [5,12,13]. These two GBM hallmarks are symbiotic, yet distinct pathobiological mechanisms of GBM therapeutic resistance [3,4,5,6,9,14]. In each case, dynamic remodeling of the actin and microtubule cytoskeletal systems generates the mechanical forces that propel cells into the extracellular matrix (ECM) [5,15]. Both mechanisms additionally require cytoskeletal plasticity to polarize cells for directed movement [16], to transport extracellular matrix (ECM) degrading enzymes to leading-edge structures [17,18], and to coordinate leading and trailing edge cellular adhesion formation, maturation, and turnover [13].

Tumor microtubes are invasive neurite-like protrusions that extend from the cell bodies of diffuse astrocytoma cells into the surrounding brain parenchyma [5]. Tumor microtube networks facilitate resistance to all three components of GBM standard of care therapy. The cellular cohort that survives radiation and chemotherapy treatment is overwhelmingly comprised of tumor microtube-connected cells [5,6]. Tumor microtubes are composed of organized arrays of microtubules and actin cytoskeleton filaments [5]. Thus, adaptable control over cytoskeletal mechanics is essential to remodel, reinforce, and maintain the long-term stability of tumor microtube networks [12,19].

Actin and microtubule reorganization is controlled, in part, by the Rho family of small GTPases. In GBM, differential activation of Rho GTPases coordinates the cytoskeletal remodeling required for invasive motility and dictates specific invasion programs [20]. Expression of the Rho GTPases Cdc42 and Rac1 is upregulated in gliomas relative to normal brain tissue [21]. Cdc42 and Rac1 activation is associated with pseudopodial extension into the brain parenchyma and guidance of other cells with lower Cdc42 and Rac1 activation towards the invasive front [20]. In U87 GBM spheroid invasions, activated Cdc42 increased migration and invasion, while Cdc42 depletion reduced invasion [22]. Rac1 inhibition similarly suppresses GBM cell invasion [23].

In contrast, RhoA’s role in GBM invasion is less clear. RhoA expression decreases with increasing grade of glial malignancy [24]. RhoA and Rac1 are known to be functionally antagonistic, with Rac1 activation predominating in mesenchymal migration and RhoA mediating the contractility required for amoeboid motility. Amoeboid motility is uncommon in GBM cells, especially in vivo. However, some RhoA activity is required for early adhesion and trailing-edge contraction in mesenchymal motility, and RhoA regulates the expression of transmembrane MMPs that remodel the ECM for mesenchymal invasion [25]. In vivo, RhoA activation is predominantly associated with perivascular invasion in vivo [20].

Rho GTPases exert their effects through the activation of downstream effector proteins [26]. Rho-associated kinases (ROCK1 and 2) and mammalian diaphanous-related formins (mDia1, 2, and 3) are major effectors for the Rho subgroup of the Rho GTPase family (RhoA-C, for instance) [27,28,29]. ROCK is a Rho family-specific serine-threonine kinase with many described functions [30], but its most well-characterized function is the coordination of actomyosin crosslinking and cellular contractility [31]. In contrast, mDia is known to additionally act downstream of Rac and Cdc42 [32,33], and nucleates and polymerizes linear actin filaments and stabilizes microtubule arrays [27,34], fundamental to the formation of cellular protrusions. Precise spatial and temporal balancing of the activity between these two effectors ultimately dictates cell shape, adhesion turnover, and motility strategies in both normal and tumor cells [35].

The effects of ROCK inhibition (ROCKi) in GBM are disputed. In some cases, ROCKi significantly decreased invasion and provided significant survival benefits in animal models of high-grade glioma [36,37]. Other times, ROCKi significantly increased invasion [38], stabilized pro-invasive tumor microtube networks, and decreased sensitivity to standard of care radiation and chemotherapy [39]. We previously demonstrated that both global activation and inhibition of mDia are effective anti-invasive and anti-tumor microtube strategies in GBM, with activation representing the superior approach [12,40]. Here, we used multiple patient-derived 3D models of *IDH*-wild-type GBMs and a novel semi-adherent in vitro model of tumor microtube networks to investigate the relationship between ROCK and mDia in GBM invasion and tumor microtube network formation.

## 2. Materials and Methods

### 2.1. GBM Patient Cell Line Isolation and Culture, Reagents, and Drugs

De-identified surgical samples were used to establish the GBM patient-derived cell lines termed Pat9, Pat27, Pat 31 parental (Pat31p), Pat31 recurrence (Pat31r), and Pat48r (therapy-resistant recurrence). These were derived from 4 separate patients. The Pat9 cell line was derived from a surgical resection from a 32-year-old Caucasian male and was *IDH1/2* wild-type. The Pat27 cell line was derived from a surgical resection from a 39-year-old Caucasian male and was *IDH1/2* wild type. Pat 31p is from a 58-year-old Caucasian female and was *IDH1/2* wild type; her recurrent tumor was resected 6 months later and was called Pat31r. Pat48r is a recurrent tumor from a 66-year-old male (NIH racial category not identified) who had previously undergone surgery, radiotherapy, temozolomide, and Optune treatments prior to recurrence 3 years and 2 months later. All participating donors gave written informed consent prior to surgical tissue collection. The study was conducted in accordance with the declaration of Helsinki, and the protocol was approved by the University of Toledo/ProMedica Hospitals Joint Institutional Review Board (IRB#201913).

De-identified surgical samples were collected from the University of Toledo Medical Center or ProMedica Toledo Hospital. Resected tumors were transported in PBS on ice. Single-cell isolation was performed as described previously [12]. Briefly, tumors were washed with D-PBS and documented with an iPhone camera. Tumors were minced with surgical scalpels. For cell isolation, a portion of minced tumors was placed in 0.05% trypsin (Gibco-Thermo Fisher Scientific, Waltham, MA, USA) and rotated at 37 °C for at least 45 min. Tumors were triturated and tissue returned to 37 °C with rotation and neutralized with DMEM/10% FBS. Cells were treated with red blood cell lysis buffer (0.15 M NH_4_Cl, 10 mM NaHCO_3_, and 0.1 mM EDTA) and centrifuged and resuspended in neural basal media (Gibco-Thermo Fisher Scientific) supplemented with 1× B27 (Gibco-Thermo Fisher Scientific), 20 ng/mL bFGF and EGF (Peprotech, Cranbury, NJ, USA), 1× sodium pyruvate, 1× GlutaMax, and 1× anti-anti (Gibco-Thermo Fisher Scientific). Cells were strained through 70 μM strainers (Thermo Fisher Scientific, Waltham, MA, USA) and plated into 6-well tissue culture plates (USA Scientific, Ocala, FL, USA). Media were changed at 24 h.

Spheres spontaneously formed in isolated patient sample monolayer cells. Spheres that had detached from monolayers were collected using wide-orifice pipette tips and moved to poly-HEMA (Millipore-Sigma, St. Louis, MO, USA) coated U-bottom 96-well plates. Spheres were used for assays upon reaching 200–250 μm in diameter. Upon reaching 350 μm, spheres were dissociated and re-plated in poly-HEMA U-bottom plates at 2000 cells/well.

To form what we termed “2.5D” semi-adherent patient-derived cell cultures, free-floating spheres were dissociated into a single-cell suspension using either 0.25% trypsin (Gibco) or Accumax Solution (Innovative Cell Technologies, San Diego, CA, USA) and mechanical trituration. Neutralized single-cell suspensions were passaged into polystyrene dishes pre-coated with 10 μg/mL type I collagen (Corning, Tewksbury, MA, USA) and 1 μg/mL fibronectin (BD Biosciences, Franklin Lakes, NJ, USA).

All cell lines were routinely screened for mycoplasma, as described [41]. IMM02 was provided as a kind gift from the late Dr. Arthur Alberts (Van Andel Research Institute, Grand Rapids, MI, USA) and Y-27362 was from Abcam (Cambridge, UK).

### 2.2. Invasion Assays, Immunofluorescence, and Microscopy

Patient-derived 3D spheroid invasion assays were as described [12]. In brief, thin layers of 5 mg/mL GFR matrigel (Corning) were pipetted in 8-well chamber glasses (Thermo Fisher Scientific). Spheres were embedded when they reached 240–260 μm in size. Spheres were added and topped with a thin layer of Matrigel. Matrigel polymerized for 45 min at 37 °C before the addition of 250 μL of media with IMMs [15,17]. Invasions were imaged at time zero (T0) and every 24 h for experimental durations. Invaded spheroids/spontaneous spheres were imaged using an EVOS inverted microscope (Advanced Microscopy Group, Bothell, WA, USA) with an Olympus 4x UplanFL N0.13 PhP objective lens. Invasive areas were measured by forming a polygon by circularly connecting vertices of the furthest invaded point in each direction and measuring the total internal area of the polygon. All measurements were calibrated to and completed with MetaMorph software (Molecular Devices, San Jose, CA, USA).

Tumor microtube lengths were measured in Metamorph using the polyline flexible line measurement, from the cell body to the vertex of each individual tumor microtube.

Cell body movement measurements were performed in Metamorph by measuring the distance from a straight line from the center of the neurosphere core to the center of the invading cell body.

Immunofluorescence staining of fixed 3D-invaded spheres was performed as previously described [12]. The following antibodies were used: mDia2 and mDia1 (1:100) or β-Tubulin (1:100) (Millipore-Sigma, Burlington, MA, USA), or Glu-Tubulin (1:100) (Millipore-Sigma), nestin (1:100) (Thermo Fisher Scientific), and GAP-43 (1:300) (Proteintech) antibodies were incubated at 4 °C for 48–72 h. Invasions were washed with PBS-T before adding AlexaFluor 2° antibodies (1:200–500), AlexaFluor Phalloidin (1:100), or DAPI (1:50) (Thermo Fisher Scientific) for 24–48 h at 4 °C.

Immunofluorescent imaging was performed on a Leica TCS SP5 multiphoton laser scanning confocal microscope. A Leica HCX PL APO 10x/0.40 CS dry or HCX PL APO 20x/0.70 CS dry UV objective lens (Leica Microsystems, Buffalo Grove, IL, USA) was used to generate Z-stack images with optical sections taken in 2.5 μm steps. Presented confocal images are projection maximum superimpositions of all optical sections for a single neurosphere. Phase contrast and bright field images were generated using an EVOS inverted microscope) equipped with an Olympus UPlanFL 4x/0.13 PhP or UPlanFL 10x/0.30 objective lens (Olympus, Center Valley, PA, USA).

### 2.3. Western Blotting and Reagents

Free-floating patient-derived 3D spheres were collected, pelleted, and resuspended in lysis buffer (0.5 M Tris-HCL pH 6.8, glycerol, 10% (*w*/*v*) SDS, and bromophenol blue supplemented with dithiotheritol (DTT)). Spheres were rotated in lysis buffer at 4 °C for 45 min. A modified Lowry method was used to quantify total lysate protein concentration (Bio-Rad Laboratories). Samples were mixed with 2X Laemmli sample buffer (Bio-Rad) and boiled for ten minutes prior to loading into gels. Electrophoresis was used to resolve proteins in a 4–20% mini-protean TGX gel before transfer to PVDF membranes using the BioRad Trans-Blot Turbo System. Membranes were blocked in 5% non-fat dry milk and probed with primary antibodies against mDia1 (Proteintech), mDia2 (Proteintech), ROCK1 (Proteintech), ROCK2 (Proteintech), and GAPDH (Proteintech). Membranes were washed with TBST, then incubated with peroxidase-conjugated secondary antibodies (Proteintech). Blots were exposed using chemiluminescence via Clarity Western ECL (BioRad) and imaged using a G:BOX2 imaging station (Syngene, Frederick, MD, USA). Densitometry analysis was performed by normalizing the chemiluminescent signal to GAPDH controls using ImageJ image analysis software. Whole-cell lysates from 2.5D patient-derived semi-adherent cultures were collected directly in lysis buffer by scraping culture dishes. Lysates were transferred to Eppendorf tubes, rotated at 4 °C for 45 min, and processed as above.

### 2.4. Statistical Analysis

Statistical analyses were performed using PRISM 9 software (GraphPad, San Diego, CA, USA). Ordinary one-way ANOVA was used to assess normally distributed data with Tukey’s range test used for post hoc analyses. A combination of the Kruskal–Wallis test and Dunn’s multiple comparison test was used equivalently for non-parametric data. Error bars reflect the standard error of the mean (SEM) across an indicated number of experimental replicates. All experiments were replicated to provide at least 80% power. For all statistical analyses, *p*-values ≤ 0.05 were considered significant.

## 3. Results

### 3.1. mDia Agonism Induces Loss of mDia Protein Expression and Is Associated with the Elimination of Tumor Microtube Networks

**Figure 1:**
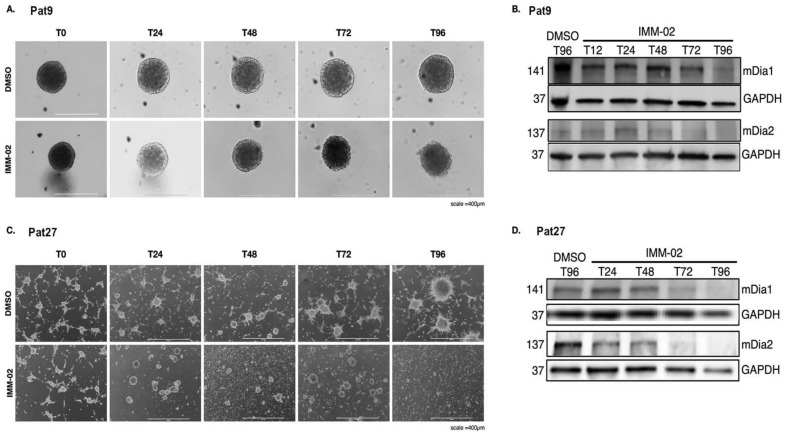
Pharmacological mDia agonism dynamically affects its expression and is accompanied by loss of GBM patient-derived sphere and tumor microtube integrity. (**A**) 10X phase-contrast images of free-floating Pat9 3D spheroids at indicated timepoints (in hours) maintained in DMSO (top) or 50 μM IMM-02 (bottom). Scale bars = 400 μm. (**B**) Western blots of cell lysates from free-floating Pat9 3D spheroids treated with DMSO or 50 μM IMM-02 at indicated time points (in h). Molecular weight markers (kDa) are listed on left. Blotting antibodies are listed on right of blot. (**C**) 4X phase-contrast images of Pat27 2.5D cultures at indicated time points maintained in DMSO (top) or 50 μM IMM-02 (bottom). Scale bars = 1000 μm. (**D**) Western blots of cell lysates from 2.5D Pat27 cultures treated with DMSO or 50 μM IMM-02 at indicated timepoints.

We previously observed that continuous pharmacological mDia2 activation with the small-molecule intramimic agonist IMM02 triggers a mesenchymal-to-amoeboid transition in invading patient-derived Pat9 GBM spheres that is associated with inhibition of new tumor microtube formation, loss of existing tumor microtubes, and a block of invasion [12]. Using a panel of patient-derived GBM primary cell lines cultured as spheres (with cell doubling times of several days), we first evaluated if mDia agonism with IMM02 equally affected invading cells derived from different patients, as well as within the same patient from matched primary and recurrent tumors (see Methods and Materials for cell line descriptions, Figure S1). Our GBM patient-derived spheroids invaded to various extents, but all showed sensitivity to IMM in invasion assays over 96 h, which was slowly recovered after drug removal, indicating a cytostatic, as opposed to cytotoxic IMM response. To investigate the mechanism by which sustained IMM02-mediated pharmacological mDia2 agonism functionally disrupts GBM tumor microtube dynamics, we examined protein expression levels in patient-derived GBM spheres treated with IMM02. Morphologically, free-floating patient-derived Pat9 GBM spheres treated with IMM02 for 96 h progressively disbanded and sphere surfaces became ragged by 72 h of treatment, relative to the smooth sphere surfaces of control DMSO-treated spheres (Figure 1A). Interestingly, this phenotypic response is well associated with mDia inhibition in our previous breast and ovarian cancer 2D and 3D spheroid models, not mDia agonism, as seen here in GBM spheres [42,43,44]. We performed Western blots of 3D sphere lysates to investigate the stability of mDia1 and mDia2 protein expression through 96 h of IMM02 treatment, which surprisingly revealed a progressive loss of both mDia1 and mDia2 protein expression in response to the extended duration of IMM02 agonist treatment, relative to control-treated cells (Figure 1B and Figure S2A), with some variations in kinetics between experiments, pointing to experimental variability and/or compensatory expression mechanisms from other mDia formins, as seen previously [32].

We then examined whether these observations could be extended to an experimental system in which GBM patient-derived cells are reinforced through a highly connected tumor microtube network. To do this, we similarly evaluated mDia1 and mDia2 protein expression dynamics through 96 h of IMM02 treatment in semi-adherent “2.5D” GBM patient-derived Pat27 cell cultures. In this experimental system, thin-layer type-I collagen and fibronectin ECM protein coatings promote robust and long-lived tumor microtube formation that interconnects large well-formed clusters of cells. We found that cell lysates can be more easily and reproducibly prepared using the “2.5D” system, relative to 3D systems, and the ultralong tumor microtube architecture is preserved. In control-treated “2.5D” Pat27 cultures, we observed a robust interconnected network of ultra-long tumor microtubes connecting well-formed clusters of GBM cells that grew in both the X, Y, and Z directional planes. Over the 96 h sustained IMM02 treatment course, we again observed a clear and progressive rounding of peripheral cells that was accompanied by dissolution of the tumor microtube network and spheres themselves (Figure 1C). These phenotypic changes similarly correlated with a progressive loss of mDia1 and mDia2 protein expression relative to controls which proved even greater in magnitude than previously observed in 3D (Figure 1D). Relative to DMSO treatment, mDia2 protein expression in Pat27 cells demonstrated the most immediate and robust decrease in response to IMM02 treatment, with 66.1% of expression remaining at the 24 h timepoint and only 2.6% detected after 96 h (Figure 1D and Figure S2B). Thus, the loss of mDia1 and mDia2 protein expression observed in response to sustained IMM02-induced global mDia agonism could be considered broadly as an “event-driven” pharmacologic strategy [45] that could reasonably be used to alter the molecular and functional cellular phenotypes in our GBM patient-derived cell line experimental system. For the bulk of the remaining experiments, we utilized Pat9 GBM spheres as they reproducibly and quickly formed compact spheres of uniform sizes for embedding into invasion assays.

### 3.2. ROCK-Directed Contractility Machinery Regulates Patient-Derived GBM Pro-Invasive Tumor Microtube Networks

**Figure 2:**
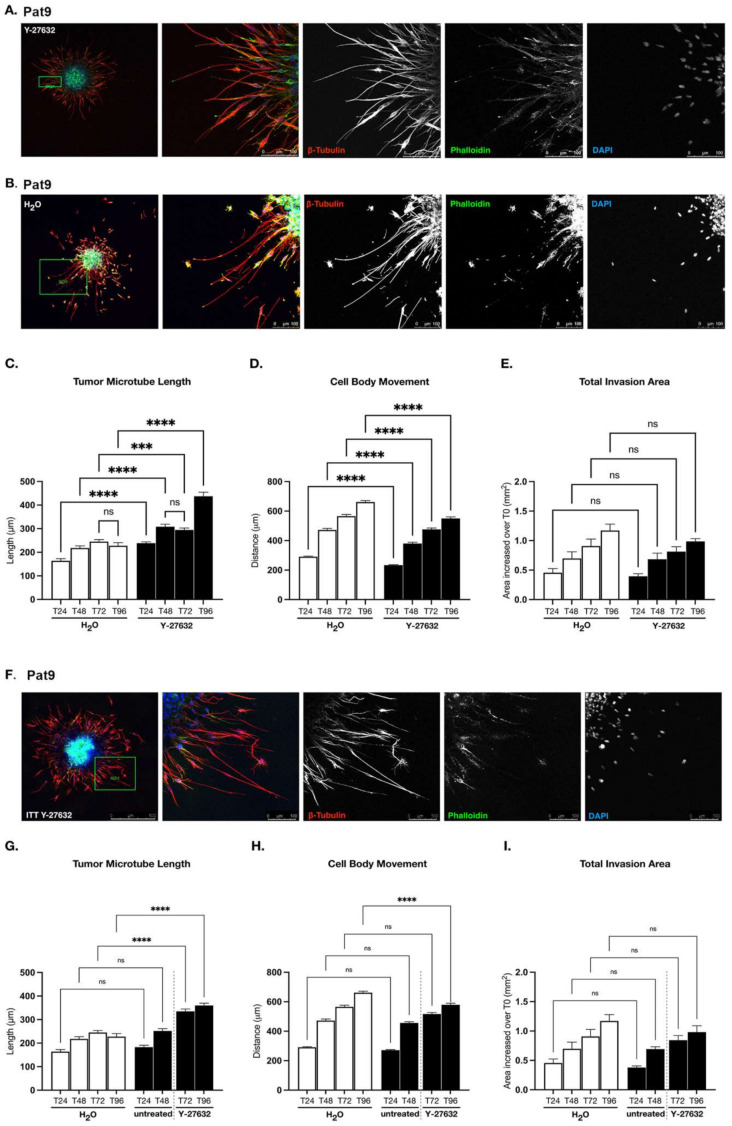
ROCK-directed contractility machinery regulates patient-derived GBM pro-invasive tumor microtube networks. (**A**) Confocal images of leading edge at T96 in fixed Y-27632-treated (90 μM) Pat9 3D invasion assay stained for β-tubulin, phalloidin, and DAPI. Scale bars = 100 μm. (**B**) Confocal images of leading edge at T96 in fixed H_2_O-treated Pat9 3D invasion assay stained for β-tubulin, phalloidin, and DAPI. Scale bars = 100 μm. (**C**) Tumor microtubule length in H_2_O- or Y-276632-treated (90 μM) Pat9 96 h 3D invasion assays. **** *p* ≤ 0.0001; *** *p* ≤ 0.001; ns = not significant. (**D**) Distance of cell body movement from the sphere core in H_2_O- or Y-276632-treated (90 μM) Pat9 96 h 3D invasion assays. **** *p* ≤ 0.0001. (**E**) Increase in total area of invasion over T0 in H_2_O- or Y-276632-treated (90 μM) Pat9 96 h 3D invasion assays. (**F**) Confocal images of leading edge at T96 in fixed Pat9 3D invade-then-treat (ITT) assays treated with Y-276632 (90 μM). Stained for β-tubulin, phalloidin, and DAPI. Scale bars = 100 μm. (**G**) Tumor microtubule length in H_2_O- or Y-276632-ITT (90 μM) Pat9 96 h 3D invasion assays. Dotted line shows time of drug introduction. **** *p* ≤ 0.0001. (**H**) Distance of cell body movement from the neurosphere core in H_2_O- or Y-276632-ITT (90 μM) Pat9 96 h 3D invasion assays. Dotted line shows time of drug introduction. **** *p* ≤ 0.0001. (**I**) Increase in total area of invasion over T0 in H_2_O- or Y-276632-ITT (90 μM) Pat9 96 h 3D invasion assays. Dotted line shows time of drug introduction. Note: (**G**–**I**) experimental procedure was performed in the same experiment/time as (**C**–**E**), but results were split onto 2 graphs for clarity. The same controls are accordingly graphed in (**G**–**I**) as in (**C**–**E**).

The balance of both distribution and activities of ROCK and mDia proteins exert a strong influence over tumor cell motility strategies and mesenchymal–amoeboid transitions [35]. To interrogate how this relationship is affected in IMM02-mediated amoeboid transitions, we first evaluated the independent role of ROCK-mediated actinomyosin contractility in Pat9 GBM sphere invasion and tumor microtube polymerization. We treated patient-derived Pat9 spheres embedded in a 3D matrix with either vehicle (water) or Y-27632 dihydrochloride, an ATP-competitive inhibitor of ROCK I/II. With vehicle treatment, embedded Pat9 GBM spheres readily invaded 3D matrices through 96 h. Confocal imaging of spheres revealed ultra-long cellular projections consistent with tumor microtubes; this was confirmed in untreated spheres by assessing the expression of markers for GBM cell and tumor microtubes, including nestin, GAP-43, tubulin, and F-actin, consistent with GBM tumor microtubes (Figure 2A,B and Figure S3). Upon ROCKi with Y-27632, Pat9 spheres invading for 96 h formed tumor microtubes that were significantly longer than those measured in vehicle-treated invasion assays (Figure 2A–C), consistent with a previous report in GBM [39]. Interestingly, while pro-invasive tumor microtubes were elongated in ROCKi cells, Pat9 cell movement as a unit independent of a cytoskeleton projection was static (Figure 2A,B, leftmost panels). That is, relative to vehicle controls, Y-27632 significantly reduced the movement of Pat9 cell bodies (measured from the center of the nucleus) in the direction of invasion, as seen with a lack of cell bodies distally invading from sphere cores. Control cell nuclei were measured having moved hundreds of microns away from sphere cores over 96 h. Thus for ROCKi cells, this effectively diminished overall cellular egress from sphere cores (as measured from the sphere center) relative to vehicle controls (Figure 2A,B (left panels) and 2D). Taken together, elongated tumor microtubes and diminished cell body movement resulted in a total invasion area (encircling the invasive front) that was comparable between vehicle-treated and ROCKi invasion assays (Figure 2E), yet the mechanisms of motility in control and ROCKi GBM cells are distinct from one another. As in Figure 1, we examined whether ROCKi via Y-27632 treatment impacted target protein expression levels. Indeed, ROCKi did not suppress ROCK2 expression, yet there was a measurable increase in ROCK1 expression—a possible compensatory mechanism for ROCKi (Figure S4A,B). mDia1 and mDia2 expression were unaffected durably at 96 h of Y-27632 treatment.

We examined if these observations could be reproduced in Pat9 spheres that were allowed to invade for 48 h before Y-27632 treatment (invade-then-treat; ITT), yielding a more clinically relevant experimental scenario. Relative to vehicle-treated controls (from Figure 2B, see Figure legend note) and within this experimental cohort, ROCKi triggered a significant increase in actin- and tubulin-enriched tumor microtube length in already invading cells (Figure 2F,G). Upon ROCKi, there were associated decreases in the motility of previously (first 48 h of the experimental timeline) invasive cell bodies relative to controls (Figure 2H). While 48 h of uninterrupted invasion facilitated substantial cell egress from sphere cores, we did not detect significant differences in the total invasion area between ITT-treated and untreated conditions at the experimental endpoint (Figure 2I). ROCK inhibition in GBM patient-derived spheres can therefore be considered to reliably constrain cell body movement at the expense of facilitating tumor microtube elongation.

### 3.3. Targeting ROCK and mDia Has Opposing Effects on Patient-Derived GBM Cell Motility and Tumor Microtube Extension

**Figure 3:**
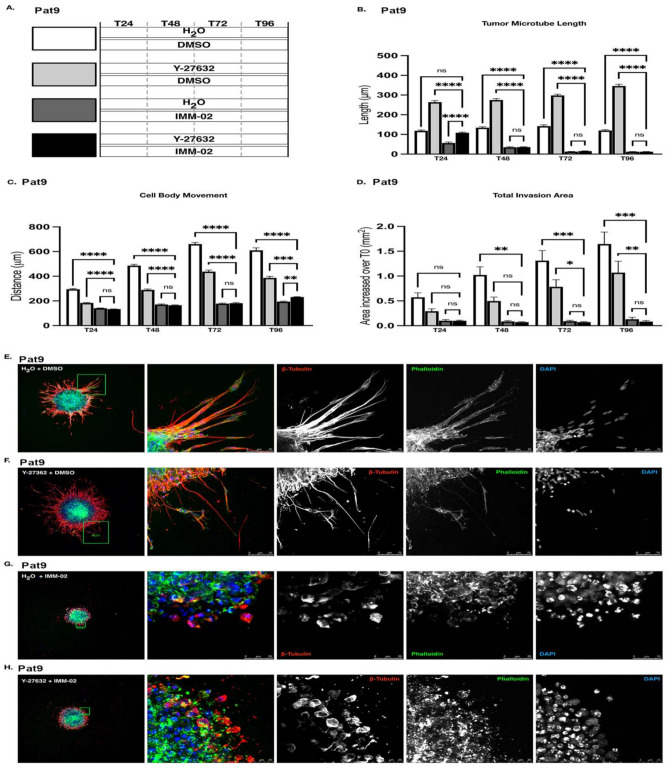
Combined targeting of ROCK and mDia halts GBM patient-derived sphere invasion, yet is not superior to mDia formin agonism alone. (**A**) Schematic of drug exposures in combination drug 3D invasion assays (Y-27632 = 90 μM; IMM02 = 50 μM). Bars at left indicate 48 h invasion prior to indicated drug treatment. (**B**) Tumor microtubule length in indicated Pat9 96 h 3D invasion assays. **** *p* ≤ 0.0001; ns = not significant. (**C**) Distance of cell body movement from the neurosphere core in indicated Pat9 96 h 3D invasion assays. ** *p* ≤ 0.01; *** *p* ≤ 0.001, **** *p* ≤ 0.0001. (**D**) Increase in total area of invasion over T0 in indicated Pat9 96 h 3D invasion assays. *** *p* ≤ 0.001; ** *p* ≤ 0.01; * *p* ≤ 0.05. (**E**) Confocal images of leading edge at T96 in fixed Pat9 3D invasion assays treated with H_2_O + DMSO. Stained for β-tubulin, phalloidin, and DAPI. Scale bars = 75 μm. (**F**) Confocal images of leading edge at T96 in fixed Pat9 3D invasion assays treated with Y-27632 + DMSO. Stained for β-tubulin, phalloidin, and DAPI. Scale bars = 75 μm. (**G**) Confocal images of leading edge at T96 in fixed Pat9 3D invasion assays treated with H_2_O + IMM02. Stained for β-tubulin, phalloidin, and DAPI. Scale bars = 25 μm. (**H**) Confocal images of leading edge at T96 in fixed Pat9 3D invasion assays treated with H_2_O + IMM-02. Stained for β-tubulin, phalloidin, and DAPI. Scale bars = 25 μm.

We showed that combined ROCKi and mDia2 inhibition is more effective at preventing invasive egress in epithelial ovarian cancer spheroids than individual targeting of either effector alone [44]. In our GBM system, mDia protein expression is lost in response to IMM02-driven mDia global activation in patient-derived GBM spheroids (Figure 1). We next investigated whether IMM02-directed mDia agonism, when combined with Y-27632, enhanced the anti-invasive effects beyond those effects observed with either drug alone (Figure 3A) [12,46]. Upon treating already invading (48 h) patient-derived Pat9 GBM spheres in 3D matrix with a combination of Y-27632 and IMM02, we observed a significant reduction in tumor microtube length in invading cells, relative to vehicle- and Y-27632-treated invasion assays (Figure 3B). Interestingly, tumor microtube length in combination-treated invasion assays was not significantly reduced in comparison to assays treated with IMM02 alone (Figure 3B). Combination treatment also diminished cell body movement of leading-edge cells and total area of invasion in comparison to vehicle-treated and Y-27632-treated invasion assays, but not in comparison to those treated with IMM02 alone (Figure 3C,D). IMM02 treatment alone proved more effective than combination treatment at inhibiting cell body invasive egress away from the sphere core relative to vehicle (Figure 3C). Relative to vehicle-treated invasion assays, Y-27632 and IMM02 combinations phenotypically disrupted the ability of Pat9 spheres to polymerize tumor microtubes in a way similar to IMM02 treatment alone (Figure 3E–H). However, the invasive edge of combination-treated spheres was notably more disorganized with a greater degree of de-adhesion than the invasive edges of spheres treated with IMM02 alone (Figure 3G,H). These findings suggest that in our GBM patient-derived spheroid invasion assays, combined treatment with IMM02 and Y-27632 is not superior to IMM02 treatment alone.

### 3.4. ROCKi Priming in GBM Cells Fails to Augment Cellular Sensitivity to mDia Agonism

**Figure 4:**
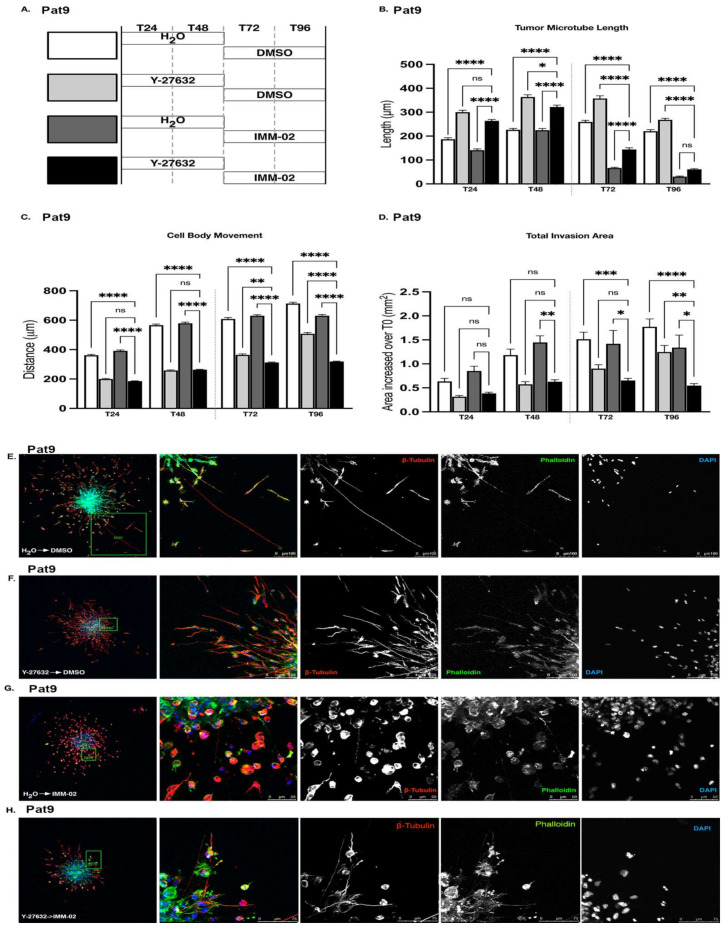
Altered the sequencing of combined ROCKi/mDia targeting does not modulate GBM invasion. (**A**) Schematic of drug exposures in drug-switch 3D invasion assays (Y-27632 = 90 μM; IMM-02 = 50 μM). (**B**) Tumor microtubule length in indicated Pat9 96 h drug-switch 3D invasion assays. Dotted line shows time of drug switch. **** *p* ≤ 0.0001; * *p* ≤ 0.05; ns = not significant. (**C**) Distance of cell body movement from the neurosphere core in indicated Pat9 96 h drug-switch 3D invasion assays. Dotted line shows time of drug switch. **** *p* ≤ 0.0001; ** *p* ≤ 0.01. (**D**) Increase in total area of invasion over T0 in indicated Pat9 96 h drug-switch 3D invasion assays. Dotted line shows time of drug switch. **** *p* ≤ 0.0001; *** *p* ≤ 0.001; ** *p* ≤ 0.01; * *p* ≤ 0.05. (**E**) Confocal images of leading edge at T96 in fixed Pat9 drug-switch 3D invasion assays treated with H_2_O-then-DMSO. Stained for β-tubulin, phalloidin, and DAPI. Scale bars = 100 μm. (**F**) Confocal images of leading edge at T96 in fixed Pat9 drug-switch 3D invasion assays treated with Y-27632-then-DMSO. Stained for β-tubulin, phalloidin, and DAPI. Scale bars = 100 μm. (**G**) Confocal images of leading edge at T96 in fixed Pat9 drug-switch 3D invasion assays treated with H_2_O-then-IMM02. Stained for β-tubulin, phalloidin, and DAPI. Scale bars = 50 μm. (**H**) Confocal images of leading edge at T96 in fixed Pat9 switch-drug 3D invasion assays treated with Y27632-then-IMM02. Stained for β-tubulin, phalloidin, and DAPI. Scale bars = 100 μm.

The order and schedule of drug administration can significantly affect therapeutic synergy [47]. To investigate if these factors impact the synergistic potential of combined mDia/ROCK targeting in our experimental system, we next administered Y-27632 and IMM02 in series rather than in parallel, as in Figure 3. Patient-derived Pat9 GBM spheres were again embedded in a 3D matrix. Invaded cells were treated with Y-27632 or vehicle for 48 h, then switched to IMM02 or vehicle treatment for an additional 48 h (Figure 4A experimental schematic). At the 96 h experimental endpoint, we did not observe a significant difference in tumor microtube length between invasion assays that were treated with a combination of Y-27632-then-IMM02 and invasions that were treated with vehicle-then-IMM02 alone (Figure 4B). In contrast to parallel administration of Y-27632 and IMM02 (Figure 3B), invasions pre-treated with Y-27632 displayed tumor microtubes that were significantly longer than invasions pre-treated with vehicle at every other experimental timepoint (Figure 4B). Invasion assays in the Y-27632-then-IMM02 experimental group also demonstrated the greatest experimental reductions in both the distance of cell body movement (Figure 4C) and the total invasion area (Figure 4D) after 96 h, relative to controls. The switch to IMM02 treatment after ROCKi in invading cells halted further cell body movement and eliminated further invasive motility irrespective of what invading cells were treated with first (Y-27632 or vehicle).

Morphologically, leading-edge cells in the Y-27632-then-vehicle cohort exhibited TMs that were distinct from control invasion assays after 96 h (Figure 4E,F). Control invasion assays formed directed and linear tumor microtubes (Figure 4E), and this morphology was consistent with observations from our experiments with Y-27632 alone (Figure 2B) and Y-27632 + vehicle in parallel (Figure 3F). In contrast, tumor microtubes in the Y-27632-then-vehicle invasion group exhibited directionality but were notably rippled along their length (Figure 4F). This phenotype was seen upon complete washout of vehicle and drug (Figure S5). Leading-edge cells from invading spheroids in the vehicle-then-IMM02 group were predominantly morphologically amoeboid at experimental endpoints (Figure 4G). By comparison, the invasive front of the Y-27632-then-IMM02 treatment group remained predominantly mesenchymal upon IMM02 exposure and marked cytoskeletal interruptions were evident in tumor microtubes after 96 h (Figure 4H). We observed fewer F-actin and β-tubulin disruptions throughout the remaining tumor microtubes of vehicle-then-IMM02-treated invasion assays; these interrupted tumor microtubes largely originated from the few cells that retained a somewhat polarized shape under these conditions (Figure 4G). Thus, tumor microtube morphological disruption associated with Y-27632 pretreatment does not sensitize invasive cells to IMM02 and may instead promote tumor microtube cytoskeletal maintenance.

### 3.5. Sustained ROCKi Delays Cellular Responses to mDia agonists in Invading Patient-Derived GBM Spheroids

**Figure 5:**
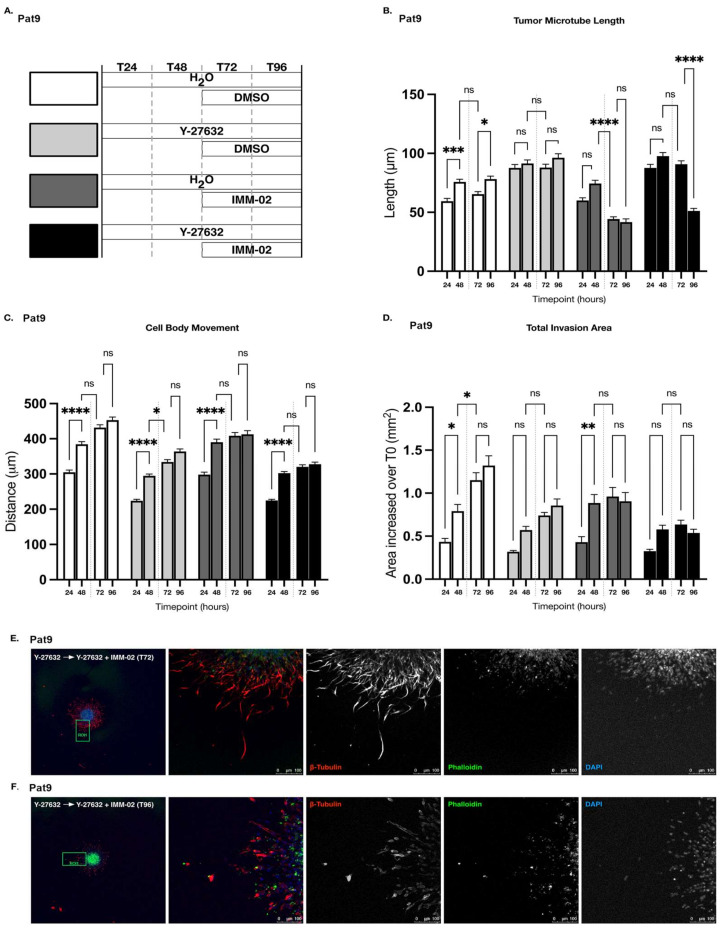
Sustained ROCKi postpones cellular responses to mDia agonists in invading GBM spheroids. (**A**) Schematic of drug exposures in add-drug 3D invasion assays (Y-27632 = 90 μM; IMM-02 = 50 μM). (**B**) Tumor microtubule length in indicated Pat9 96 h add-drug invasion assays. Dotted line shows time of drug addition. **** *p* ≤ 0.0001; *** *p* ≤ 0.001; * *p* ≤ 0.05; ns = not significant. (**C**) Distance of cell body movement from the sphere core in indicated Pat9 96 h add-drug invasion assays. Dotted line shows time of drug addition. **** *p* ≤ 0.0001; * *p* ≤ 0.05. (**D**) Increase in total invasion area over T0 in indicated Pat9 96 h add-drug 3D invasion assays. Dotted line shows time of drug addition. ** *p* ≤ 0.01; * *p* ≤ 0.05. (**E**) Confocal images of leading edge at T72 in Pat9 add-drug 3D invasion assays treated with Y-27632-then-(Y-27632 + IMM-02). Stained for β-tubulin, phalloidin, and DAPI. Scale bars = 100 μm. (**F**) Confocal images of leading edge at T96 in fixed Pat9 add-drug 3D invasion assays treated with Y-27632-then-(Y-27632 + IMM-02). Stained for β-tubulin, phalloidin, and DAPI. Scale bars = 100 μm.

IMM02-driven mDia agonism induced patient-derived GBM cell tumor microtube collapse in both invasion assays pre-treated with Y-27632 and those pre-treated with vehicle. However, the rate of tumor microtube collapse (mean reduction in length/day) was significantly slower in invasions pre-treated with Y-27632 than in those pre-treated with vehicle (Figure 4B). We, therefore, hypothesized that ROCK activity could be required for the IMM02-mediated collapse of tumor microtubes. To address this question, we pre-treated already invading 3D spheres with Y-27632 for 48 h, but instead of performing drug washout and switching to IMM02 after pre-treatment, we added IMM02 while maintaining Y-27632 for an additional 48 h of combination treatment (Figure 5A). This allowed for sustained ROCKi through the addition of IMM02. Such pre-treatment with Y-27632 followed by combination treatment with Y-27632 and IMM02 delayed the IMM02-mediated reduction in tumor microtube length to an even greater degree than pre-treatment with Y-27632 alone. Within 24 h of IMM02 addition to vehicle-treated invading cells, there was significantly reduced tumor microtube length (−40.5%), while adding IMM02 to Y-27632-treated invasions insignificantly reduced tumor microtube length by only 7% (Figure 5B). Still, the difference in tumor microtube length remained insignificant between the vehicle and Y-27632 pre-treated cohorts at the 96 h experimental endpoint. Interestingly, the effects of sustained Y-27632 exposure did not similarly delay the IMM02-mediated reduction in cell body movement (Figure 5C) or the prevention of further significant increases in total invasion area (Figure 5D). Individual cells at the invasive front displayed morphology that mirrored these quantifications—cells remained mesenchymal in shape at the 72 h timepoint (24 h after the addition of IMM02) (Figure 5E) but were far more compact and amoeboid after 96 h (48 h after the addition of IMM02) (Figure 5F). Collectively, these findings support a role for ROCK in pro-invasive tumor microtube dynamics but highlight a complex interplay between ROCK and mDia proteins in maintaining pro-invasive tumor microtubes.

## 4. Discussion

In this study, we provide evidence that diffuse CNS invasion and tumor microtube network formation in GBM rely on balancing the antagonistic activities of mDia and ROCK. We demonstrated that persistent pharmacological activation of mDia using IMM02—a small molecule mDia agonist—indirectly disrupts the function of mDia in multiple patient-derived models of *IDH*-wild-type GBM. Pharmacological disruption of ROCK activity prevented invasive cell body movement away from the spheroid core but induced the formation of ultra-long and phenotypically abnormal tumor microtubes in a 3D patient-derived model of GBM invasion. When combined with IMM02 in a variety of dosing schedules, ROCK inhibition did not enhance the previously described anti-invasive and anti-tumor microtube effects of IMM02 treatment [12]. In contrast, ROCK inhibition delayed and lessened the magnitude of the IMM02-mediated collapse of invasive tumor microtubes.

We used growth-factor reduced (GFR) Matrigel for our invasion assays for our GBM patient-derived spheres. While Matrigel is not a perfect model matrix or brain ECM because it is enriched in laminin and collagen, GFR Matrigel also has heparin sulfate proteoglycans which are a component of intraparenchymal brain ECM. While imperfect, other factors support the use of GFR Matrigel to study tumor microtubes. For instance, others have found that laminin may be a component of the ventricular zone, subplate, and marginal zone of the developing cerebral wall, and that laminin expression along routes of migrating neurons implies that glial laminin may serve as a substratum for neuronal attachment [48,49,50]. Additionally, the tumor microtube phenotype is more associated with intraparenchymal invasion pattern, not perivascular invasion pattern in vivo, suggesting that GFR Matrigel sufficiently models this niche [51].

GBM cells predominantly rely on a proteolysis-guided mesenchymal or collective pattern of motility [13,52,53]. Wherein active mDia drives extension of leading-edge protrusions (such as tumor microtubes) [54], active ROCK coordinates trailing edge detachment and retraction [52]. In our experimental system, ROCK inhibition induced changes consistent with these described roles. Previous studies in GBM show that targeting mDia activation [12,46] or preventing proteolytic ECM degradation [55] can induce a mesenchymal-to-amoeboid transition in which invasive cells abandon directed mesenchymal migration in favor of a ROCK-dominant contractility-based motility strategy [56]. This phenomenon also occurs in other systemic cancers, where combined targeting of proteolysis or mDia and ROCK blocked both the mesenchymal and amoeboid modes of motility and proved superior to targeting either motility pathway alone [44,57]. Here, we again observed a mesenchymal-to-amoeboid transition upon introduction of IMM02 [12] but found that combination targeting of these two Rho effectors was inferior to targeting mDia alone.

The universal superiority of IMM02 in our experiments may be explained by our observation that IMM02 indirectly disrupts the function of mDia. In multiple patient-derived models of *IDH*-wild-type GBM, IMM02 consistently and progressively induced the loss of mDia1 and mDia2 protein expression over a 96 h experimental time course. Though we previously showed that IMM-mediated mDia pharmacological activation is superior to direct SMIFH2-mediated mDia inhibition as an anti-invasion and anti-tumor microtube strategy in multiple models of GBM, these findings may offer a rudimentary mechanistic explanation for why both mDia targeting strategies noticeably inhibit GBM invasion and tumor microtube formation [12,46]. There is a precedent for proteosomic elimination [58] and small-molecule-mediated loss of mDia protein expression in other systems [59]. To the best of our knowledge, this is the first reported incidence of a small-molecule agonist inducing the endogenous destruction of multiple mDia formins. Thus, we propose that IMM02 could be conceptualized as an activator of endogenous mDia inhibition in GBM, by acting in an event-driven mode of pharmacological suppression of protein expression.

The observed effects of IMM02 treatment in GBM models are better understood by examining the consequences of mDia loss. mDia function is essential to maintaining and remodeling both the actin and microtubule cytoskeletal systems. These systems support diffuse CNS invasions, tumor microtube formation, and long-term tumor microtube network stability. Consequently, GBM invasive motility is consistently and significantly affected by mDia knockdown [54,60,61,62,63]. We and others also showed that GBM invasion and tumor microtubes are highly sensitive to nocodazole and other novel microtubule targeting agents [12,19], but it remains to be determined whether these processes are similarly affected by isolated inhibition of actin dynamics. Profound mDia loss may also explain why the IMM02-induced mesenchymal-to-amoeboid morphological transition is not associated with a switch to amoeboid motility [12]. ROCK is traditionally considered to be a master regulator of cellular contraction and amoeboid motility, but mDia-dependent actin polymerization is required to retract the non-apoptotic blebs that drive amoeboid motion [42,64]. Loss of this function would prevent amoeboid motility and is consistent with the robust non-apoptotic blebbing and subsequent bleb rupture that we observe upon high-powered microscopic examination of IMM02-treated invasion assays (unpublished observation). mDia formins are also required for the maturation and turnover of focal adhesions [54], orientation of the microtubule-organizing center, mitotic chromosomal segregation [65], and they essentially participate in the maintenance of the stem phenotype in both GBM [66] and normal neural progenitor cells [67].

It is of note to point out that ROCK inhibition consequently leads to changes in cellular tension that ultimately could impact cell–cell interactions or cellular engagement with extracellular matrix (ECM) components. Likewise, mDia interrogation in other 3D sphere systems impacted cell–cell adhesions [44]. In our recent work in ovarian cancer monolayers, altering mDia formin function using genetic or pharmacodynamics approaches altered sphere integrity via modifying cell–cell junctions and 2D-junctional integrity while concurrently reducing vinculin and junctional protein localization to junctions, indicating changes in junctional tension [68]. Interestingly RhoA-directed ROCK has been shown to regulate the activity of gap-junctional protein Connexin-43 in neuronal cells and in brains in vivo [69]. As Connexin-43 is an integral protein in tumor microtube dynamics in GBM cells [5], it would be interesting to further understand in future studies whether altering mDia or ROCK activities impacts tumor microtubes and/or cell-cell/ECM junction via targeting connexin-43 expression.

Correspondingly, mDia formins embody many similar roles in normal neurodevelopmental processes where mDia loss is clinically associated with microcephaly [70,71] and experimentally linked to defects in axonogenesis, neural progenitor migration, dendritogenesis, and synaptogenesis [67,72,73,74]. While mDia silencing is linked to increased metastasis and invasion in some systemic cancers, these CNS-specific roles for mDia mitigate concerns for a similar phenomenon in GBM. A reasonable and related question remains of whether IMM02 may induce the collapse of stable adult axons. We did not observe increased cell death upon IMM02 treatment in an ex vivo rat brain slice model of GBM invasion [46], nor did we observe neurodevelopmental abnormalities in embryonic zebrafish treated with IMM02 [40]. However, this possibility has not yet been ruled out and experiments are currently underway to examine the effects of IMM02 on in vitro cultures of IPSC neurons.

## 5. Conclusions

Despite the clinical availability of FDA-approved ROCK inhibitors, these findings support previous assertions that ROCK inhibition is not a viable therapeutic strategy in GBM because it enriches the pro-invasive tumor microtube network and thereby likely facilitates the development of chemo and radioresistance in these tumors [39]. In contrast, IMM02—a small molecule mDia agonist—prevents the formation and maintenance of GBM tumor microtubes and prevents both mesenchymal and amoeboid invasive motility.

## Data Availability

Not applicable.

## Supplementary Materials

The following supporting information can be downloaded at: https://www.mdpi.com/article/10.3390/cells11091559/s1, Figure S1: Multiple glioblastoma patient-derived cell lines are sensitive to IMM02-mediated sphere invasion suppression; Figure S2: Densitometry of Figure 1 blots; Figure S3: Immunofluorescence validation of tumor microtube markers; Figure S4: Evaluating protein expression changes in Y-27632-treated cells; Figure S5: Y-27632 drug washout impacts upon spheroid invasion.
