# Supplementary material for: The Cytoskeleton Effectors Rho-Kinase (ROCK) and Mammalian Diaphanous-Related (mDia) Formin Have Dynamic Roles in Tumor Microtube Formation in Invasive Glioblastoma Cells
Source: Cells. 2022 May 5;11(9):1559. doi: 10.3390/cells11091559 (PMC9103681; doi:10.3390/cells11091559)
Supplement: Supplementary file 1 [file cells-11-01559-s001.zip › cells-1643048-supplementary.pdf]

cells

MDPI

Supplementary data

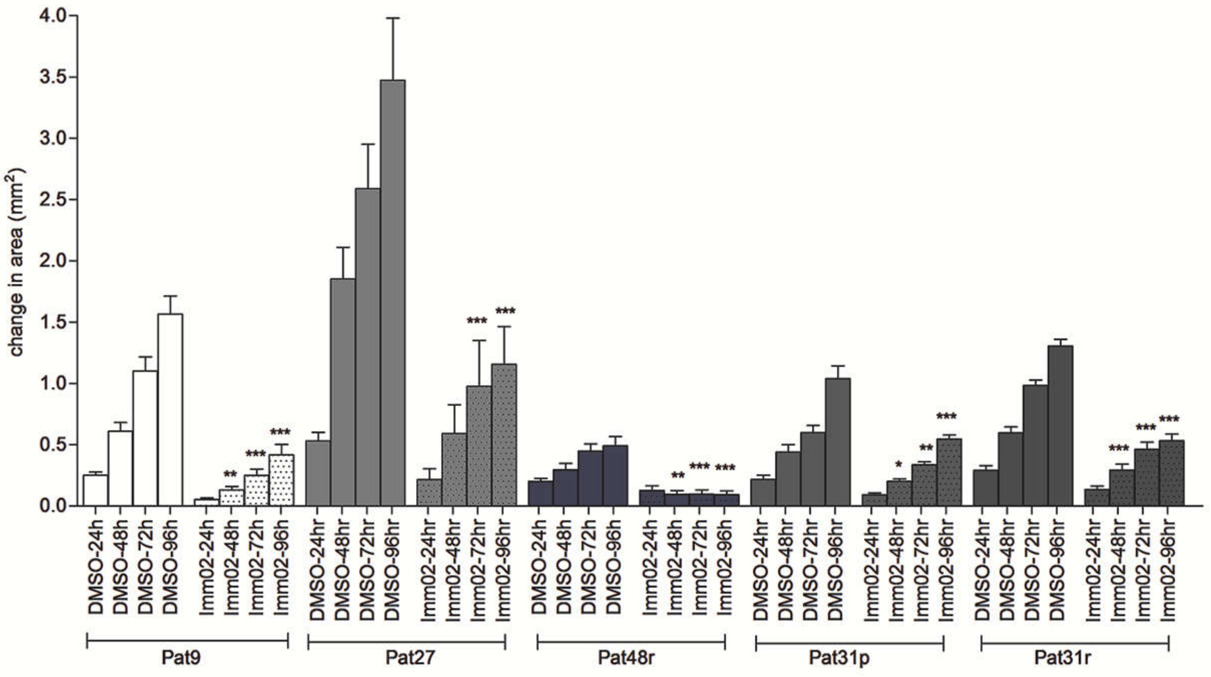

Figure S1. Multiple glioblastoma patient-derived cell lines are sensitive to IMM02-mediated sphere invasion suppression. Pat9, Pat 27 and Pat31p were derived from primary tumors. Pat31r is the matched recurrent tumor to Pat31p. Pat48r is a non-matched recurrent tumor that failed 4 4 therapies (see Methods). Spheres of approximately 250-280mm were embedded in matrigel and concurrently treated with 50mM IMM02. After 24h, IMM was washed away and cells invaded for an additional 72h. ***p<0.001. Error bars indicate standard error. Shown is at least 3 replicate experiments with each condition performed at least in triplicate.

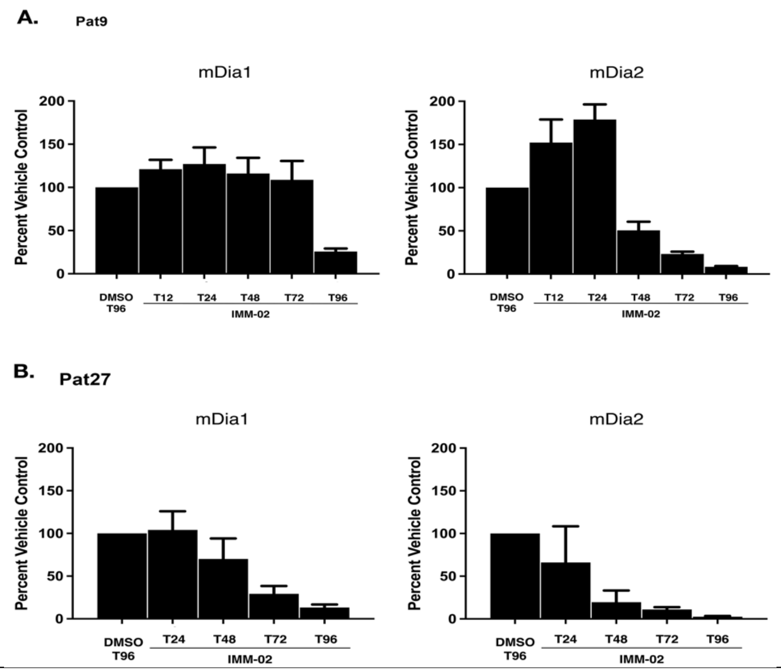

Figure S2. Densitometry of Figure 1 blots. **A.,** Densitometry of western-blots of lysates from free-floating Pat9 3D spheres treated with DMSO or 50 μM IMM-02 at indicated timepoints. Represenative blots of at least three repeats shown in **Figure 1B**. B., Densitometry of western-blots of lysates from 2.5D Pat27 cultures treated with DMSO or 50 μM IMM-02 at indicated timepoints. Representative blots of at least three repeats shown in **Figure 1D**.

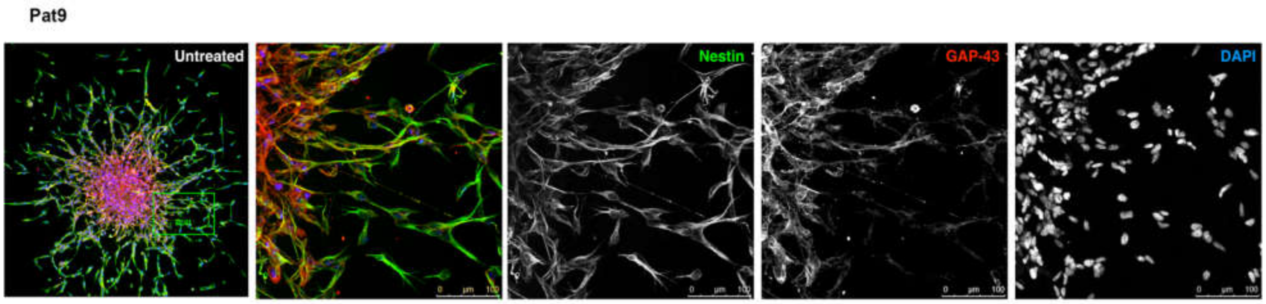

Figure S3. Immunofluorescence validation of tumor microtube markers. Confocal images of leading edge at T96 in fixed untreated Pat9 3D invasion assay stained for nestin, GAP-43, and DAPI. Scale bars = 100 μm.

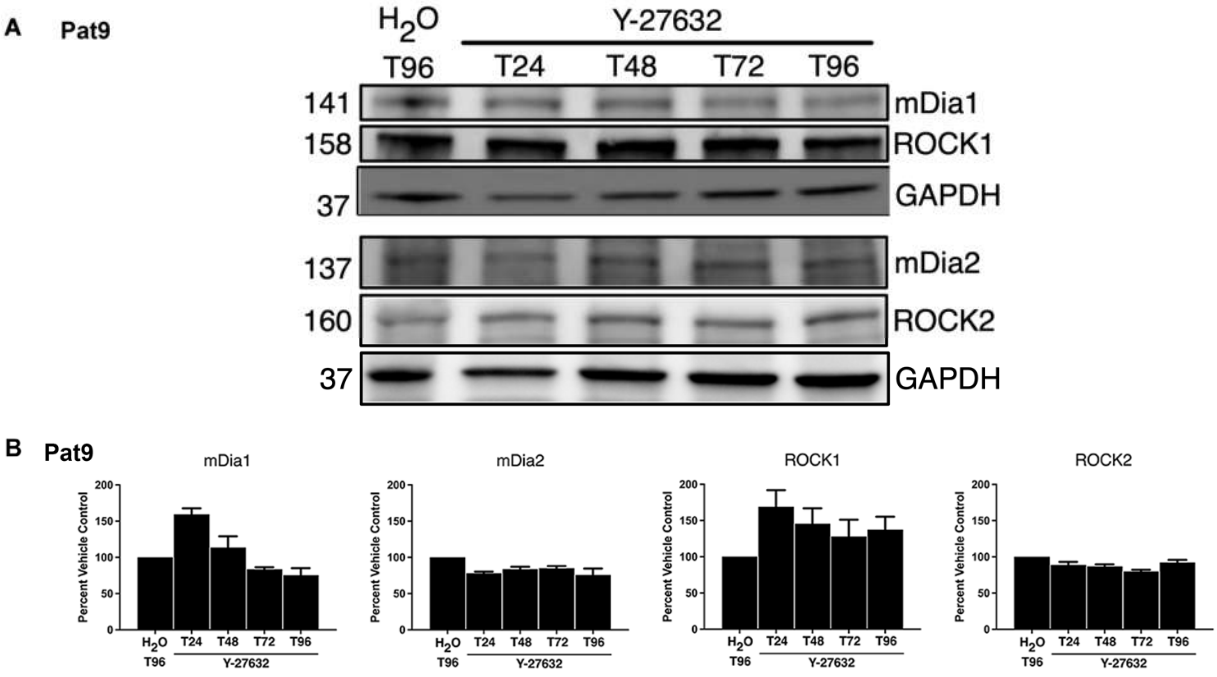

Figure S4. Evaluating protein expression changes in Y-27632-treated cells. **A.,** Western-blots of lysates from free-floating Pat9 3D spheres treated with H_2O or 90 μM Y-27632 at indicated timepoints. **B.,** Densitometry of western-blots of lysates from free-floating Pat9 3D spheres treated with H_2O or 90 μM Y-27632 at indicated timepoints. Densitometry shown is calculated from at least three experiments. Error bars represent standard error.

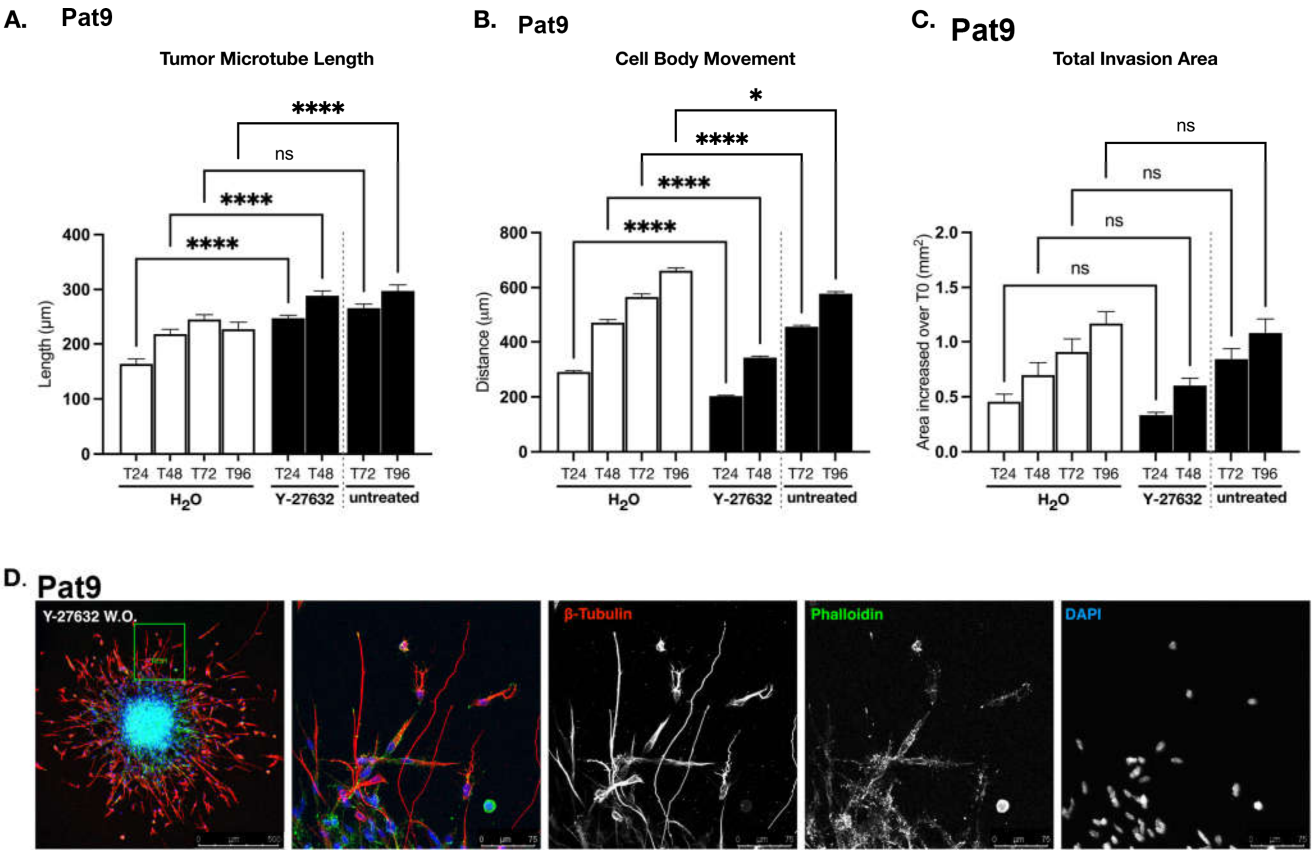

Figure S5: Y-27632 drug washout impacts upon spheroid invasion. A., Tumor microtubule length in indicated Pat9 96-hour H_2O- or Y-27632-treated (90 μM) drug-washout 3D invasion assays. Dotted line shows time of drug washout. ****p≤0.0001; ns = not significant. **B.,** Distance of cell body movement from the sphere core in indicated Pat9 96-hour H_2O- or Y-27632-treated (90 μM) drug-washout 3D invasion assays. Dotted line shows time of drug washout. ****p≤0.0001; *p≤0.05. **C.,** Increase in total area of invasion over T0 in indicated Pat9 96-hour H_2O- or Y-27632-treated (90 μM) drug-washout 3D invasion assays. Dotted line shows time of drug washout. ns = not significant. **D.,** Confocal images of leading edge at T96 in fixed Pat9 Y-27632-treated (90 μM) drug-washout 3D invasion assays. Stained for β-tubulin, phalloidin, and DAPI. Scale bar total sphere = 500 μm. Scale bars ROI = 75 μm.
